# Implementation of Fluorescent-Protein-Based Quantification Analysis in L-Form Bacteria

**DOI:** 10.3390/bioengineering11010081

**Published:** 2024-01-15

**Authors:** Di Tian, Yiyuan Liu, Yueyue Zhang, Yunfei Liu, Yang Xia, Boying Xu, Jian Xu, Tetsuya Yomo

**Affiliations:** Laboratory of Biology and Information Science, School of Life Sciences, East China Normal University, Shanghai 200062, China

**Keywords:** cell-wall-less bacteria, L-form, heterogeneity, quantification analysis, fluorescent protein labeling

## Abstract

Cell-wall-less (L-form) bacteria exhibit morphological complexity and heterogeneity, complicating quantitative analysis of them under internal and external stimuli. Stable and efficient labeling is needed for the fluorescence-based quantitative cell analysis of L-forms during growth and proliferation. Here, we evaluated the expression of multiple fluorescent proteins (FPs) under different promoters in the *Bacillus subtilis* L-form strain LR2 using confocal microscopy and imaging flow cytometry. Among others, P*_ylb_*-derived *NBP3510* showed a superior performance for inducing several FPs including EGFP and mKO2 in both the wild-type and L-form strains. Moreover, *NBP3510* was also active in *Escherichia coli* and its L-form strain NC-7. Employing these established FP-labeled strains, we demonstrated distinct morphologies in the L-form bacteria in a quantitative manner. Given cell-wall-deficient bacteria are considered protocell and synthetic cell models, the generated cell lines in our work could be valuable for L-form-based research.

## 1. Introduction

Cell-wall-less (L-form) bacteria can grow and reproduce after long-term adaption to various stressors, such as inhibitors of peptidoglycan biosynthesis, high temperature and nutrient starvation [1,2,3]. The L-forms induced from rod-shaped bacteria, e.g., *Escherichia coli* and *Bacillus subtilis*, exhibit spherical or pleomorphic shapes due to the lack of cell walls that maintain their original morphology [1,4]. It was described that the shape of the *E. coli* L-form strain NC-7 varies between spherical, angular and cylindrical [5], with intracellular vesicles and small membrane particles easily detectable [4]. Therefore, quantitative analysis is more challenging for L-forms due to their high complexity and heterogeneity, unlike the wild type with intact cell walls.

Advanced microscopy and flow cytometry (FCM)-based platforms have been extensively utilized for quantitative cell analysis, both at the single-cell and population levels. The resulting high-resolution microscopic cell images can be further processed using analytical tools like ImageJ, which offers various convenient ready-to-use plugins [6,7]. Additionally, fluorescence-activated cell sorting (FACS) and imaging flow cytometry (IFC) have been developed for applications including cell sorting [8,9,10], quantification [11,12], bacterial viability assessment [13,14], dynamic monitoring of bacterial morphology [15,16] and analysis of the heterogeneity in bacterial communities [16,17]. However, the quality and reproducibility of the results of these tools primarily rely on the employed fluorescent labeling markers for targeted cells [18,19]. Fluorescent proteins (FPs) can be expressed in specific subcellular locations, enabling effective cell tracing and imaging without extra staining procedures, potential cell toxicity and the insufficient selectivity of the commercially available dyes [18,20,21,22].

A robust promoter, either constitutive or inducible, is critical for efficient protein expression in host cells, including bacteria [23]. The promoters used in *E. coli* and *B. subtilis* have been extensively studied for fundamental and practical purposes, rendering them the most employed bacterial models [24]. For example, the available options in *B. subtilis* include inducible promoters (e.g., P*_spac_*, P*_xylA_*, P*_sacB_*) [25,26,27,28,29], constitutive promoters (e.g., P_43_, P*_veg_*, P*_shuttle-_*_09_) [30,31,32], auto-inducible promoters (e.g., P*_srfA_* and P*_ylb_*) [33,34,35,36,37] and phase-specific promoters (e.g., P*_rpsF_* and P*_aprE_*) [38,39]. Auto-inducible promoters allow for protein expression without inducers, making them ideal for industrial applications while mitigating the risks associated with prolonged chemical inducer exposure (e.g., arabinose, xylose, maltose and IPTG) [36,40].

Although FP labeling in wild-type bacteria has been well investigated, only limited reports have described the expression of fluorescent proteins in cell-wall-less (L-form) strains. Currently, only green fluorescent protein (GFP) and mCherry have been tested in *B. subtilis* L-form strains as fluorescent labels or fusion proteins for studying gene function and regulation [41,42,43]. Furthermore, most of the available promoter activities remain unexplored in a cell-wall-less scenario. Thus, L-form bacteria with stable and efficient FP expression are highly valuable for quantitative cell analysis in growth and proliferation, particularly as models for synthetic cells or protocells [44].

In this study, we performed a small-scale screening for multiple FPs (GFP+, EGFP, Staygold, mKO2, mCherry, DsRed and tdTomato) expressed under different promoters (P*_spac_*, P_43_, P*_veg_* and *NBP3510*) in the *B. subtilis* L-form strain LR2 [45]. The results indicated that *NBP3510* performed better in driving fluorescent gene expressions in a growth-phase-dependent manner compared to the other tested promoters. Both the wild-type and L-form strains exhibited brighter fluorescent signals for EGFP and mKO2, among others. Surprisingly, we also identified a significant promoter activity for *NBP3510* in both *E. coli* MG1655 and the L-form strain NC-7, emphasizing its broad applicability. Quantitative analysis of the constructed fluorescent strains was conducted using microscopic and IFC techniques under various culture conditions, providing valuable insights for future investigations of L-form bacteria as early life form and synthetic cell models.

## 2. Materials and Methods

### 2.1. Bacterial Strains

The *B. subtilis* wild-type 168CA and *E. coli* wild-type K-12 MG1655 were maintained in our laboratory. The *B. subtilis* L-form LR2 strain (168CA; P*_xyl_*-murE ispA*) was gifted by Dr. Jeff Errington’s laboratory [45]. The stable L-form *E. coli* NC-7 derived from *E. coli* K12 3301 was originally obtained by Onoda et al. [46] and was gifted by Dr. Akinobu Oshima (Shimane University, Matsue, Japan).

### 2.2. Culture Conditions

The *B. subtilis* wild-type (168CA) bacteria were grown on nutrient agar (NA, Oxoid) or nutrient broth (NB, Oxoid). The *B. subtilis* L-form LR2 bacteria were cultured in an osmoprotective medium (NB/MSM) composed of 2× magnesium–sucrose–maleic acid (MSM: 40 mM MgCl_2_, 1 M sucrose, 40 mM maleic acid, pH 7.0) mixed 1:1 with 2× nutrient broth (NB, Oxoid) or 2× NA. Equally, C minimal medium (70 mM K_2_HPO_4_, 30 mM KH_2_PO_4_, 25 mM (NH_4_)_2_SO_4_, 0.5 mM MgSO_4_, 10 μM MnSO_4_, 22 mg/L ferric ammonium citrate and 50 mg/L tryptophan) was also used to culture the *B. subtilis* strains as indicated in [47]. The *E. coli* MG1655 was grown in Luria–Bertani (LB) broth (1% tryptone, 0.5% Yeast Extract, 170 mM (1%) NaCl). In the case of NC-7, the osmoprotective MLB medium containing 340 mM NaCl (1% peptone, 0.5% Yeast Extract, 30 mM glucose, 340 mM NaCl, 1 mM CaCl_2_, 25 mM MOPS pH 7.0, 100 U/mL PenG) was used for static culture without shaking [5]. All the bacteria except NC-7 were incubated at 37 °C with shaking (200 rpm) while the NC-7 cells were incubated at 30 °C statically without shaking. A final concentration of 0.5 mM or 2 mM IPTG was added to the culture medium when necessary. The antibiotics were added at the following final concentrations: 100 μg/mL Ampicillin, 25 μg/mL Chloramphenicol, 150 μg/mL Spectinomycin and 200 μg/mL D-cycloserine.

### 2.3. Plasmid Constructions

As illustrated in Figure S1A, the integrative vector PSG1154 (Ampicillin used for selections in *E. coli*, Spectinomycin used for selections in *B. subtilis*) was chosen as the backbone to be integrated into the chromosomal *amyE* locus of *B*. *subtilis* [48]. The IPTG-inducible vector P*_spac_*-*gfp*^+^ was constructed by replacing the original promoter P*_xyl_* and fluorescent protein on plasmid PSG1154 with the DNA fragment containing the P*_spac_*-*gfp*^+^-*lac*I sequence cloned from pMUTIN-*gfp*^+^ [49]. The sequences of three promoters, P*_veg_*, P_43_ and NBP3510 (Figure S2), were synthesized (Sangon, Shanghai, China) and then cloned into plasmid PSG1154 together with *gfp*^+^ to yield P*_veg_*-*gfp*^+^, P*_P_*_43_-*gfp*^+^ and *NBP3510*-*gfp*^+^. Furthermore, we chose another two green fluorescent proteins (EGFP and Staygold), three red fluorescent proteins (mCherry, DsRed and tdTomato) and one orange fluorescent protein (mKO2) to construct multiple expression vectors by replacing the *gfp*^+^ of the NBP3510-*gfp*^+^ using the Gibson assembly method. To construct the recombinant plasmid pNBP3510-mKO2_*E. coli*, three fragments from the plasmid pUC19 (*Ori*), pETcoco1 (*CmR*) and the mKO2 expression cassette were amplified using the primer pairs (listed in Table S1), and then assembled to produce a new plasmid (Chloramphenicol resistance) suitable for the *E. coli* strains. All the correct expression plasmids after DNA sequencing (Sangon, Shanghai, China) were transformed into the indicated bacterial strains. The positive fluorescent signals were confirmed using direct observation on plate and confocal microscopy (Nikon C2plus, Yokohama, Japan). The plasmid sequence data have been submitted to the GenBank database under accession number OR754211–OR754221.

### 2.4. Microplate Reader Measurement

The bacterial cells after overnight culture were diluted to the same optical density (OD) with fresh culture medium. Then, 200 μL of the diluted cells was transferred into a 96-well plate for continuous culture. The OD (λ = 600 nm) and fluorescence intensity (mKO2/mCherry/DsRed/TdTomato: λ excitation = 560 nm, λ emission = 580–611 nm; GFP+/EGFP/Staygold: λ excitation = 485 nm, λ emission = 500–530 nm) were monitored using a Multimode microplate reader (BioTek Instruments, Winooski, VT, USA; model: Synergy H1) every 0.5 h. All the samples were prepared in at least triplicate. The data were exported upon the completion of the data acquisition.

### 2.5. Confocal Microscopy Observation

The bacterial cells, which were cultured until the late logarithmic phase, were dropped onto a microscope slide and covered with a coverslip. Then, the cell samples were observed, and images were acquired using a confocal microscope (Nikon C2 plus, Yokohama, Japan). The excitation beam for mKO2/mCherry/DsRed/tdTomato was set at 561 nm and the emission signal for mKO2/DsRed/tdTomato was captured at 560–595 nm, while the λ emission for mCherry was 600–650 nm. As for GFP+/EGFP/Staygold, λ excitation = 488 nm and λ emission = 500–550 nm were used. The image analysis was performed using the NIS-ELEMENTS C-ER software (Nikon, Yokohama, Japan). All the fluorescent images acquired using the confocal microscope were captured and processed using identical parameters.

### 2.6. Imaging Flow Cytometry (IFC) Analysis

All the strains were cultured in corresponding medium until the late logarithmic phase and were analyzed using an Amnis™ ImageStream™X MK II imaging flow cytometer with the INSPIRE™ acquisition software v.201.1.0.744 (Luminex, Austin, TX, USA). Red fluorescent signals were induced with a 20 mW/200 mW 561 nm laser, and the emission was detected in Channel 3 with a 560–595 nm filter (mKO2/DsRed/tdTomato) and in Channel 4 with a 595–642 nm filter (mCherry). The green fluorescent signals were induced with a 200 mW 488 nm laser, and the emission was detected in Channel 2 with a 480–560 nm filter. The bright field and side scatter (SSC) data were collected in Channel 4/1 and Channel 6 (785 nm), respectively. For each sample, at least 3 × 10^4^ cells were acquired at 40× magnification, a pixel size of 0.25 μm^2^, a low flow rate and high sensitivity, and the measurement data were analyzed using the IDEAS analysis software (v.6.2.183.0, Luminex, Austin, TX, USA). The FlowJo software (v10.6, BD Biosciences, Ashland, OR, USA) was employed to analyze the single-cell data generated using the IFC for various parameters, including the fluorescent intensity, aspect ratio, length and area.

## 3. Results

### 3.1. Evaluation of the Fluorescent Protein Expressions in B. subtilis WT and LR2

In this study, we successfully constructed a range of plasmids bearing different constitutive promoters (P*_veg_* and P_43_), an auto-inducible promoter (*NBP3510*) and an IPTG-inducible promoter (P*_spac_*) (Figure S2). As shown in Figure S1B,C, we initially observed the colors of positive colonies from each fluorescent strain with the correct chromosomal integrations (single copy) of the *B. subtilis* WT and LR2 on solid plates (15 h culture) to evaluate the fluorescent protein expression. The green/red colors were only noticeable in the *NBP3510* promoter-containing WT strains, *NBP3510*-*egfp* and *NBP3510*-*mKO2* (Figure S1B, arrowed). While not observed in LR2 at 15 h (Figure S1C), similar green/red colonies appeared after 2 days longer of incubation. Accordingly, the cell pellets from the liquid culture of *NBP3510*-*egfp/mKO2* also exhibited apparent colors for both the WT (Figure S1D) and LR2 (Figure S1E) strains. Since P*_spac_* activation requires IPTG, further cultivation of the P*_spac_*-*gfp*^+^-transformed strain was performed on a solid plate with 0.5 mM IPTG (Figure S1F–H). Significant color changes were observed in *E. coli* DH5α (positive control, high copy number of plasmid), but neither *B. subtilis* strain showed a detectable color. Collectively, these results indicated the superiority of NBP3510 over the other promoters for EGFP and mKO2 protein expression in *B. subtilis*.

**Figure 1:**
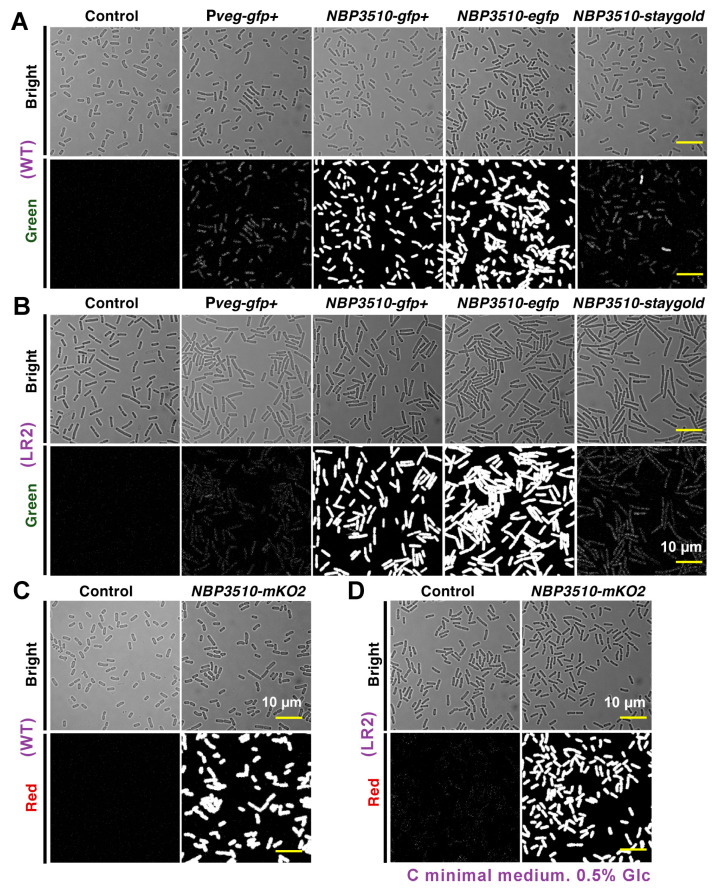
Comparison of expression levels of FPs in *B. subtilis* WT and L-form LR2. Microscopy images of strains with different promoters and FPs in *B. subtilis* WT (**A**,**C**) and L-form LR2 (**B**,**D**). The **top panel** for each strain shows bright field and the **bottom panel** shows green/red fluorescence field. All strains were cultured to late logarithmic phase in C minimal medium (CMM) and images were taken. Scale bar = 10 µm.

To directly compare the FP expression levels, we subjected late exponential phase cells to confocal microscopy to visualize the fluorescent signals. Note that the C minimal medium with a defined composition was used for both strains to avoid unexpected disturbance from the nutrient broth medium. All the cell images were obtained under consistent experimental settings, as described in Materials and Methods. The highest GFP+ fluorescence intensity was observed under *NBP3510*, in contrast to the weaker signals from the other promoters (Figure S3A,C). EGFP and mKO2 showed a superior fluorescent performance compared to the other FPs including GFP+, Staygold, mCherry, DsRed and tdTomato (Figure S3B,D). Both the *B. subtilis* WT and LR2 strains displayed similar expression levels and fluorescent intensity patterns (Figure 1), indicating the successful development of several fluorescent strains for cell quantification assays.

### 3.2. Fluorescent Protein mKO2-Based Quantification Analysis in B. subtilis WT and L-Form Cells

**Figure 2:**
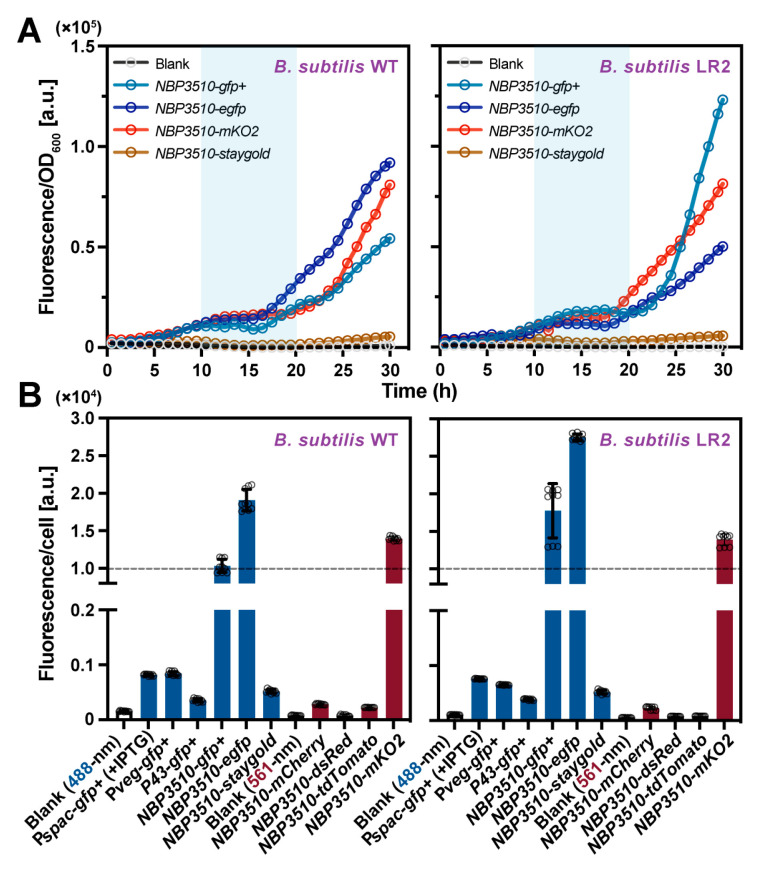
Quantitative analysis of FPs in *B. subtilis* WT and L-form LR2. (**A**) The expression levels over time were measured using a microplate reader in *B. subtilis* WT and L-form LR2 at the cell population level. (**B**) Mean fluorescent intensity from 1 × 10^4^ cells was analyzed using IFC in *B. subtilis* WT and L-form LR2 at single-cell level. All cultures were grown in triplicate, and each experiment was performed at least three times (*n* = 9). Data are mean ± standard deviation.

We then used fluorometry to quantitatively examine the dynamics of the expressed FPs during bacterial growth in the constructed cell lines. As summarized in Figures S4 and S5 and Figure 2A, the results suggested that the GFP+, EGFP and mKO2 induced by the promoter *NBP3510* effectively indicate the bacterial growth in the *B*. *subtilis* WT and LR2 cultured in the minimal medium, while the low-expressed FPs poorly indicate growth in the other strains (Figure S5A). Moreover, the expression level per OD (fluorescence intensity/OD) dramatically increased after the exponential phase (10–20 h, blue area) in all the tested strains, which is consistent with the previous result that *NBP3510* is a strong promoter during the stationary phase (Figure 2A) [36,37]. Meanwhile, the average fluorescence intensity at the single-cell level from the late exponential phase was also quantified using IFC. The bacterial cells expressing GFP+, EGFP or mKO2 exhibited an average intensity greater than 1 × 10^4^/cell (dashed line), which was significantly higher (>10-fold) than that of the other fluorescent strain cells (Figure 2B). Direct observation under a microscope and quantitative analysis at both the population and single-cell levels confirmed *NBP3510* induces relatively high-level EGFP and mKO2 expression in single-copy status without additional inducers. This characteristic makes it an ideal indicator for the *B. subtilis* WT and L-form bacteria LR2.

**Figure 3:**
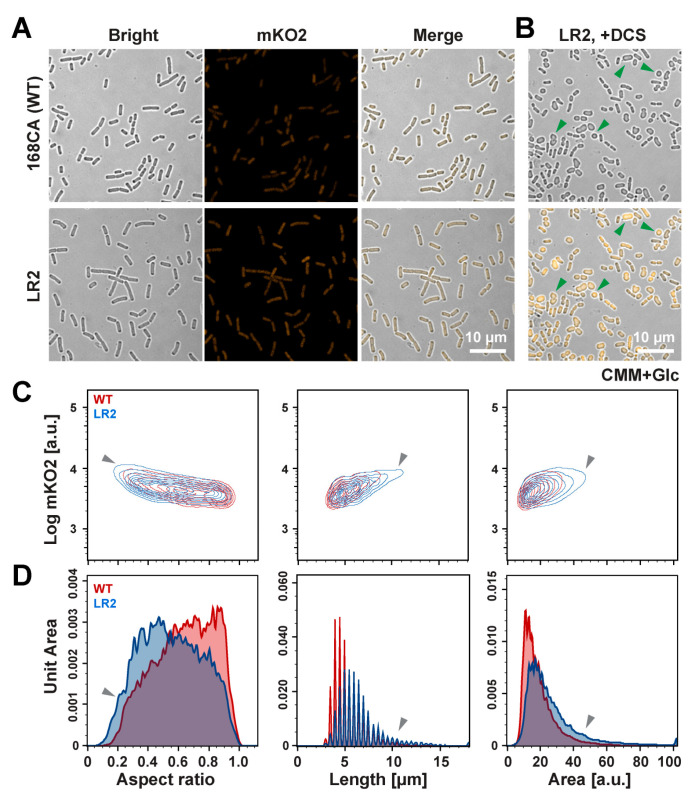
Quantitative analysis of morphology in *B. subtilis* WT and L-form LR2. Microscope images of *B. subtilis* 168CA WT ((**A**), **upper panel**) and L-form LR2 grown in C minimal medium ((**A**), **lower panel**) with or without 200 μg/mL D-cycloserine (DCS) (**B**) at late logarithmic phase. Scale bar = 10 µm. Green arrowheads indicate several typical cells with spherical shapes or undergoing irregular cell divisions. Detailed morphological changes and mKO2 expression in LR2 were analyzed using IFC and are shown in (**C**,**D**). The contour plot (contour levels: 10%) and distribution were performed for aspect ratio ((**C**)/(**D**): **left panels**), length ((**C**)/(**D**): **middle panels**) and area ((**C**)/(**D**): **right panels**). Gray arrowheads indicate the cell populations with a smaller aspect ratio, longer length and bigger area.

Both LR2 and its WT strain display a similar short rod-shaped morphology in the minimal medium (Figure 3A), which is inconsistent with the typical spherical morphology of most of the L-form bacteria in the previous reports [50]. We speculate this discrepancy may have been caused by the nutrient deprivation environment in the CMM or the residual cell walls remaining in the LR2. It is known that D-cycloserine (DCS) can efficiently induce the L-form transition in many bacteria by inhibiting cell wall lipid II precursor synthesis [50]. We then examined the morphological changes in the mKO2-labeled LR2 cells cultured in the CMM medium with DCS (Figure 3B). The direct observations under the confocal microscope reveal that LR2 can grow and exhibits more typical small spherical cells and irregular division (Figure 3B, green arrowhead). It is worth noting that the cells cultured in NB/MSM showed a remarkable diversity of shapes, regardless of DCS addition. Surprisingly, there appears to be a correlation between the higher mKO2 fluorescence level and the presence of DCS, as enhanced fluorescence was observed (Figure S6A), presumably implying that DCS may affect promoters or other unknown intracellular targets [51].

Taking advantage of the mKO2 fluorescence marker, we utilized the IFC platform to distinguish LR2 from other non-cell impurities. We then performed statistical analysis on key characteristics such as the fluorescence, cell aspect ratio, length and area. The results from the cells (*n* > 5000) showed significant differences in the aspect ratio between the DCS treatment and control groups of LR2, suggesting a shift toward a spherical shape in the bacterial morphology (Figure S6B,C). Although it is extremely difficult to differentiate between the two cell strains, the IFC analysis showed that more LR2 cells are longer, with a lower aspect ratio and larger area (gray arrowheads, Figure 3C,D), suggesting a change in bacterial elongation direction in the LR2 population. The IFC results confirm the distinct morphological changes in the LR2 cell populations in minimal medium, even though these changes were not discernible in the microscopic images.

### 3.3. NBP3510 Promoter Is Also Active in E. coli Strains

**Figure 4:**
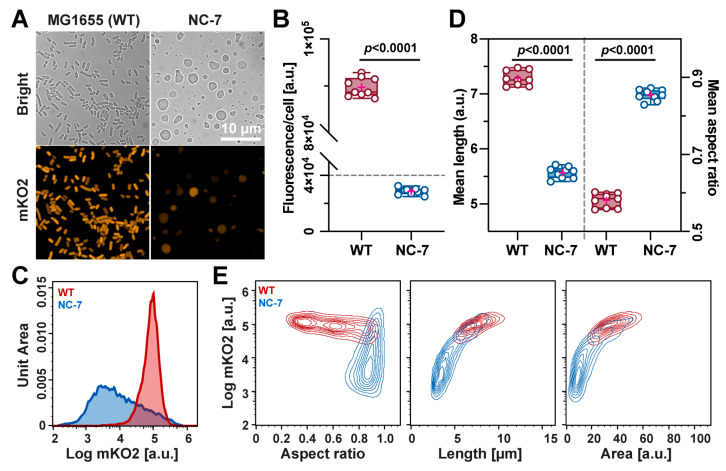
Quantitative analysis of morphology in *E. coli* MG1655 and L-form NC-7. Microscope images of fluorescent cells with p*NBP3510-mKO2* from *E. coli* MG1655 and NC-7 are shown in (**A**). Scale bar = 10 µm. IFC analysis of *E. coli* MG1655 and L-form NC-7 was performed to compare the fluorescent intensity (per cell) (**B**), mean length ((**D**), **left panel**) and mean aspect ratio ((**C**), **right panel**). All cultures were grown in triplicate and each experiment was performed at least three times (*n* = 9). Data are mean ± standard deviation. * *p* < 0.0001 (unpaired two-sided Student’s *t*-test). Red +: mean. The distributions of the mKO2 intensity in WT and NC-7 cells were compared (**C**). The contour plot (contour levels: 10%) was performed for aspect ratio ((**E**): **left panel**), length ((**E**): **middle panel**) and area ((**E**): **right panel**).

During the plasmid preparation of p*NBP3510*-*fp* for *B. subtilis*, we unexpectedly discovered that *E. coli* DH5α positive colonies showed recognizable colors (Figure S7A), indicating that *NBP3510* is also recognizable by *E. coli*. To further explore this promoter’s potential in *E. coli*, we designed a new plasmid (p*NBP3510*-*mKO2*-*Ecoli*) assembled from three fragments containing *Ori* (pMB1) of pUC19, CmR of pETcoco1 and the *NBP3510*-*mKO2* expression cassette (Figure S7B). The positive transformants in *E. coli* WT MG1655 and L-form NC-7 were then cultured in LB and MLB media, respectively. Compared to the control groups lacking the corresponding plasmid, the resulting bacterial cell pellets of each positive strain exhibited a detectable pink color, while NC-7 displayed a weak pink color, as shown in Figure S7C. To further visualize the cells, we gained fluorescent images using the confocal microscope (Figure 4A), which are consistent with the results in Figure S7C. The expression levels in NC-7 also varied notably among cells in the whole population. The fluorescence per OD in NC-7 showed a lower increase rate in mKO2 fluorescence during the exponential phase (Figure S5B), presumably due to low viability and significant differences in the plasmid copy numbers at the single-cell level in L-form cells.

### 3.4. Quantification Analysis in the E. coli WT and NC-7 L-Form Cells

The results of the nine replicates showed that the MG1655 cells had a much higher mKO2 fluorescence intensity than the NC-7 cells (Figure 4B,C). Further examination of the IFC data in Figure 4D unveiled that the MG1655 cells were longer (~6.84 μm) than the NC-7 cells (~4.86 μm), although many giant cells were present in the microscopic images (Figure 4A, right panel). The aspect ratio of the MG1655 cells (~0.57) was smaller than that of the NC-7 cells (~0.85), indicating NC-7 bacteria are more spherical. The detailed distributions of the aspect ratio, length and area versus the expressed fluorescence intensity are depicted in Figure 4E, demonstrating that NC-7 cells are spherical (aspect ratio) and contain more smaller (length and area) cells in the population. Collectively, the quantitative results from the IFC correlate well with the microscopy observations, confirming that constructed fluorescent NC-7 cells can be further applied in fluorescence-based quantitative analyses under various conditions.

## 4. Discussion

In this work, different promoters and FPs were chosen to achieve fluorescence labeling for the *B. subtilis* WT and L-form strains. While the integrated expression mode can address the possible plasmid loss during cell divisions, it requires stronger promoters to compensate for the lower expression levels. The *NBP3510* promoter is a strong stationary phase promoter [37], showing significantly higher activity than the other three promoters (P*_spac_*, P*_veg_* and P_43_) tested in this study. The best performance was observed with GFP+, EGFP and mKO2 among the FPs chosen, validated using confocal microscopy, a plate reader and IFC (Figure 1, Figure 2 and Figures S1–S5). While other FPs have been shown to be highly photostable in previous studies, such as Staygold and mCherry [42,43,52], their expressions tested in our experiments were unsatisfactory, which could be attributed to the codon bias or protein stability, or other differences in the plasmid constructions [53,54,55,56,57]. Future investigation could improve their expressions using further codon optimization and reconsiderations in the plasmid reconstruction.

Moreover, we found that the *B. subtilis* promoter *NBP3510* exhibits excellent activity in *E. coli* strains, suggesting a possible application as a universal promoter for large-scale protein productions in an inducer-free manner [37]. In general, most promoters show strain-specificity [58], with few functioning in heterologous hosts, such as P*_srfA_* [59]. *NBP3510* is an enhanced version of P*_ylb_*, created by replacing the −35, −10 core region and upstream sequence (UP), with consensus sequences [36,37]. These alterations may explain why this promoter is recognizable and drives strong gene expression in *E. coli*, though the detailed mechanism remains unknown.

Unexpectedly, we found in this study that *B. subtilis* LR2 can grow directly in CMM medium without additional osmotic stabilizers (Figure 1B). It is known that L-forms can proliferate better only when osmoprotective conditions are present [5,60]. Unlike commonly used nutrient broths like NB/MSM, CMM has a defined low-complexity composition ideal for synthetic biology applications [61]. Based on the fluorescent marker mKO2, a quantitative analysis was conducted to characterize the morphological changes in the LR2 cells in CMM in a quantitative manner. The results indicated that the LR2 cells exhibited a lower growth rate when cultured in the CMM medium (Figure 2A), potentially due to the inadequate nutritional supply in the culture environment. It is believed that cell elongation is driven by an FtsZ-independent mechanism, requiring excess membrane synthesis to generate an unbalanced surface-area-to-volume ratio, promoting cell division [45]. In environments with sufficient nutrition (NB/MSM), L-form bacteria are able to synthesize an adequate membrane from rich resources, allowing for the formation of extruded division morphologies. We hypothesize L-form growth is mostly hindered in nutrient-deficient environments like the CMM medium used in our experiments, and the typical L-form traits are less prominent than in rich medium.

Similarly, the mKO2 marker driven by *NBP3510* was applied in *E. coli* to monitor the protein expression and cell morphology. The IFC analysis showed that the NC-7 L-form was statistically rounder and smaller than the wild-type (Figure 4D). NC-7 also showed significantly lower mKO2 expression than MG1655, indicating that the L-form bacteria have a higher tendency to lose plasmids and maintain only a low copy number. The heavy mutations in NC-7, including essential genes, make it a valuable resource for minimal genome research [5,62]. To study the gene function and essentiality in NC-7 and other L-forms, it is crucial to develop a plasmid DNA transformation method for gene knockout or overexpression. However, we encountered a poor transformation efficiency and failed to establish an efficient gene manipulation platform for NC-7, highlighting the need for future efforts on NC-7-based genetics and applications. Considering the cell-wall-less status of L-form cells, it might be interesting to test whether a liposome-based DNA transfection approach is usable to improve the transformation efficacy. L-form bacteria have been already found in plants, recurrent urinary tract infections and the human tumor microbiome, implying their clinical and environmental significance [63,64,65]. The methods and plasmids generated in our research are valuable for studying the L-form bacteria in clinical and environmental samples.

## 5. Conclusions

Based on small-scale screening of various promoters and fluorescent genes, we validated a strong promoter NBP3510, which induced satisfactory gene expressions in both *B. subtilis* and *E. coli*. Both the WT and cell-wall-less bacteria were successfully labeled with the fluorescent mKO2 protein and employed for quantitative analysis of the cell morphology in two L-form bacteria models. The findings provided insights into the L-form cell behavior in different environments, offering new opportunities for using L-forms as synthetic cell models.

## Data Availability

All supporting data are included within the main article and its Supplementary Files.

## Supplementary Materials

The following supporting information can be downloaded at https://www.mdpi.com/article/10.3390/bioengineering11010081/s1, Figure S1: Plasmid construction and direct observation of expressed FPs in *B. subtilis* WT and L-form LR2; Figure S2: The sequence of different promoters used in this study; Figure S3: Microscope images of all constructed fluorescent strains; Figure S4: Growth curve of all strains used in this study; Figure S5: The expression of fluorescent protein mKO2 in *B. subtilis* WT, L-form LR2*, E. coli* MG1655 and L-form NC-7; Figure S6: Quantitative analysis of morphology in *B. subtilis* L-form LR2 with DCS; Figure S7: Construction and analysis of fluorescent expression strains of *E. coli* MG1655 and L-form NC-7; Table S1: List of primers used in this study.
