# Supplementary material for: Implementation of Fluorescent-Protein-Based Quantification Analysis in L-Form Bacteria
Source: Bioengineering (Basel). 2024 Jan 15;11(1):81. doi: 10.3390/bioengineering11010081 (PMC10813599; doi:10.3390/bioengineering11010081)
Supplement: Supplementary file 1 [file bioengineering-11-00081-s001.zip › bioengineering-2806668-supplementary.pdf]

Supplemental Figures (S1−S7) and Table S1

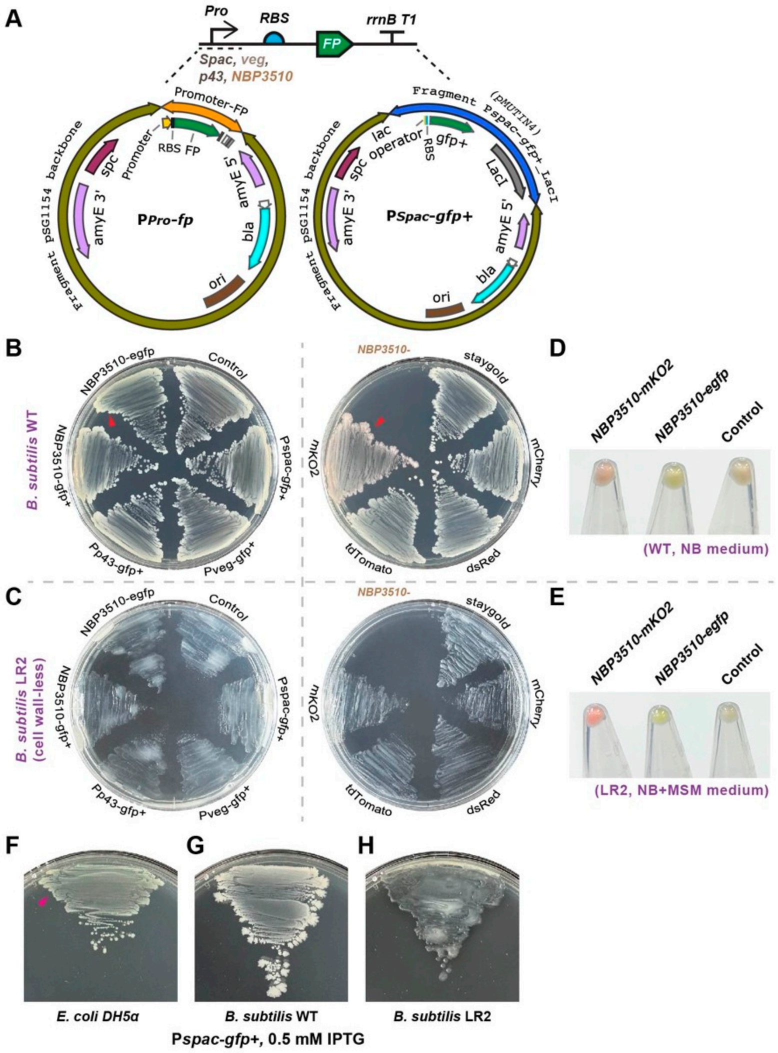

Figure S1. Plasmid construction and direct observation of expressed FPs in B. subtilis WT and L-form LR2. (A) Construction of the fluorescent protein (FP) expressing vectors (Ppro-fp and Pspac-gfp+) under various constitutive promoters (Pro: Pveg, P43, and NBP3510) and IPTG-inducible promoter Pspac. (B) All generated FP-expressing strains of B. subtilis WT (B) and LR2 (C) were grown on NA plate or NA/MSM plate, respectively. The red arrow represents apparent red or green colony in the NBP3510-egfp or NBP3510-mKO2 WT strain. Cell pellets from indicated strains cultured in liquid medium were also collected for comparisons (D&E). Comparison of different fluorescent strains with Psapc-gfp+ in the presence of IPTG (F−H). (F) E. coli DH5α strain containing Psapc-gfp+ was grown on LB plate with 0.5mM IPTG, the red arrowhead indicates the apparent green color. B. subtilis WT (G) and LR2 (H) strain containing Psapc-gfp+ was grown on NA or NA/MSM plate with 0.5 mM IPTG, respectively.

P*spac*: **ttgtgagcggataacaat**taagctt*aaggaggt*
P*veg*: **aattttgtcaaaataatttattgacaacgtcttattaacgttgatataatttaaa**
ttttatttgacaaaaatgggctcgtgttgtacaataaatgttactagag*aaaggtggtgaa*
P*p43*: **attttacatttttagaaatgggcgtgaaaaaaagcgcgcgattatgtaaaatataa**
tactagag*aaaggtggtgaa*
NBP3510: **acttctcaaagatcccatttaaaaatttttttaaaaaaatatttgacattttaa**
ataaagcgtttataatatatgtagaaacaacaaaggggagatttgtttgacaaag
gtagaacgt*gattaactaataaggaggacaaac*

Promoter sequence
RBS sequence

Figure S2. The sequence of different promoters used in this study. The promoter and RBS sequences were marked with underline and italics, respectively.

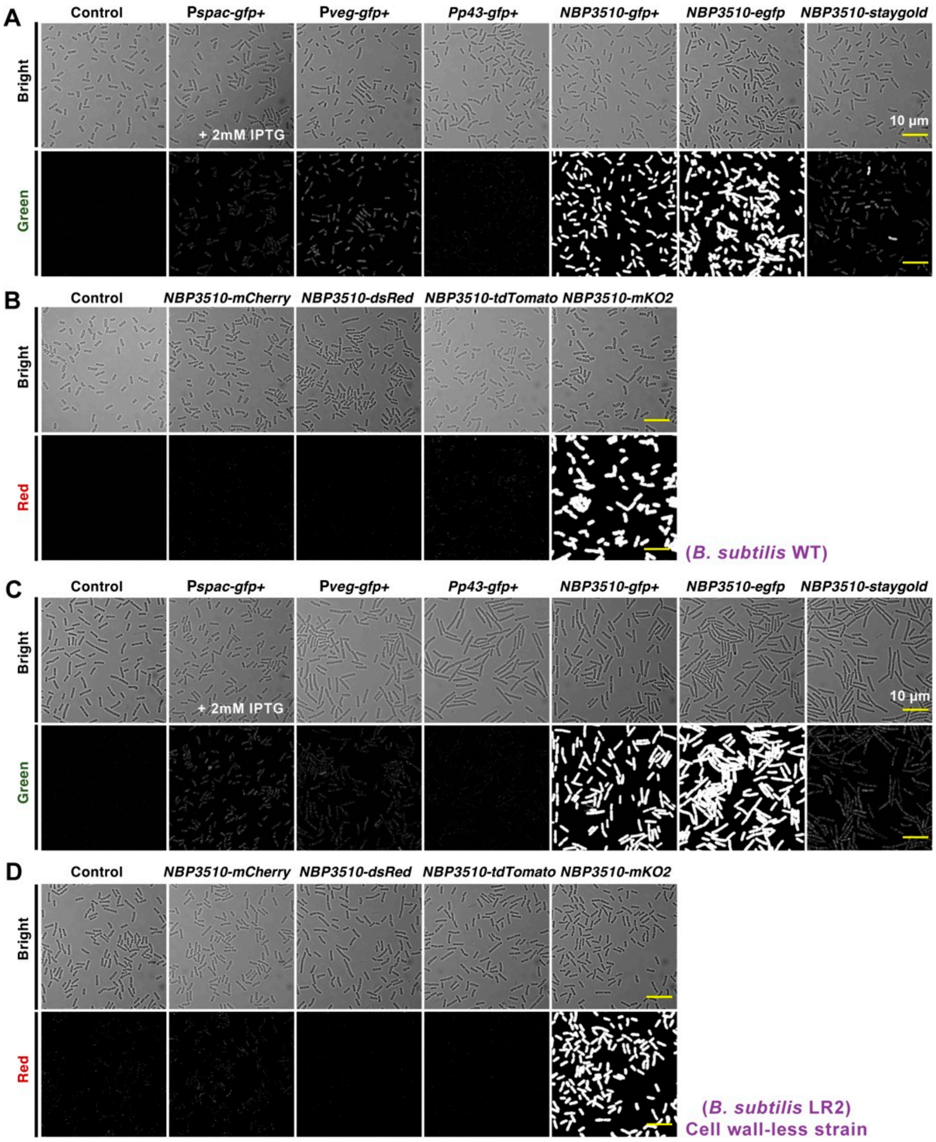

Figure S3. Microscope images of all constructed fluorescent strains. Images of tested green (A) and red (B) fluorescent strains in B. subtilis WT. Images of all tested green (C) and red (D) fluorescent strains in B. subtilis LR2. All bacteria were grown to late logarithmic phase in C minimal medium (CMM). Scale bar = 10 μm.

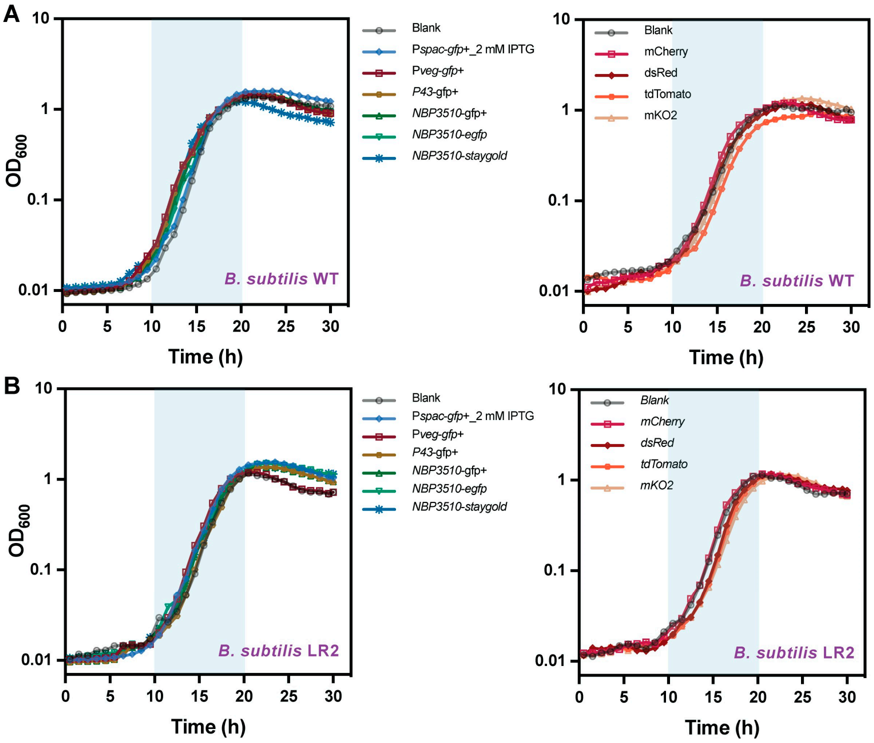

Figure S4. Growth curve of all strains used in this study. The growth curve (OD600) for all stains expressing green (left panel) or red (right panel) fluorescent proteins in B. subtilis WT (A) and L-form LR2 (B) were shown.

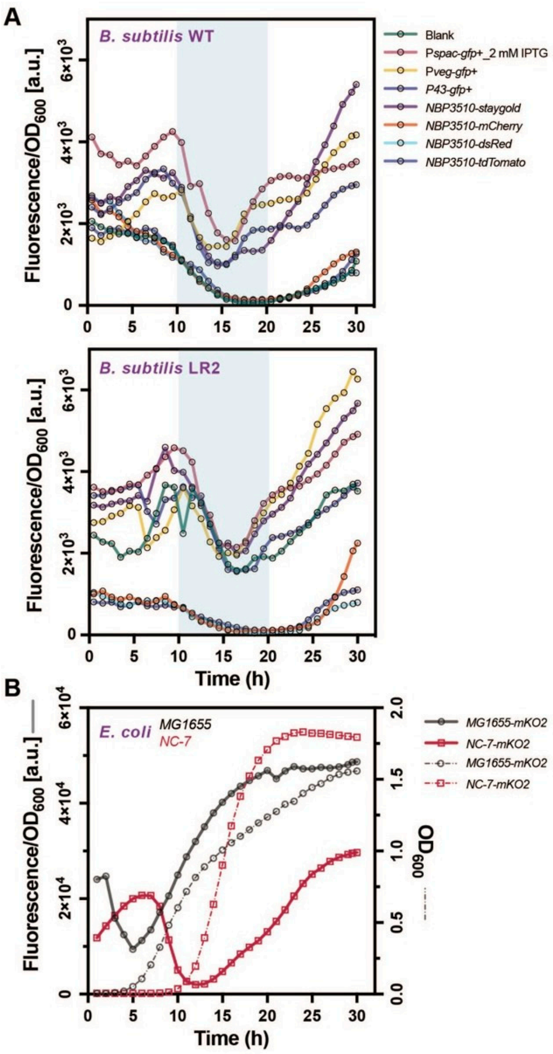

Figure S5. The expressions of fluorescent proteins in B. subtilis WT, L-form LR2, E. coli MG1655 and L-form NC-7. (A) The fluorescent expression level and pattern measured by the microplate reader in B. subtilis WT (upper panel) and L-form LR2 (lower panel). (B) The fluorescent expression level and growth pattern in E. coli MG1655 and L-form NC-7.

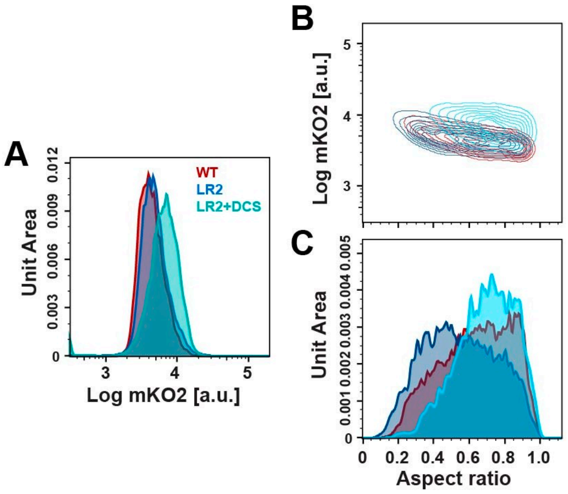

Figure S6. Quantitative analysis of morphology in B. subtilis L-form LR2 with DCS. The distributions of mKO2 intensity in cells from WT, LR2 (-), and LR2 (200 μg/mL DCS) were compared and plotted in (A). The contour plot (contour levels: 10%) (B) and distribution (C) were performed for the aspect ratio.

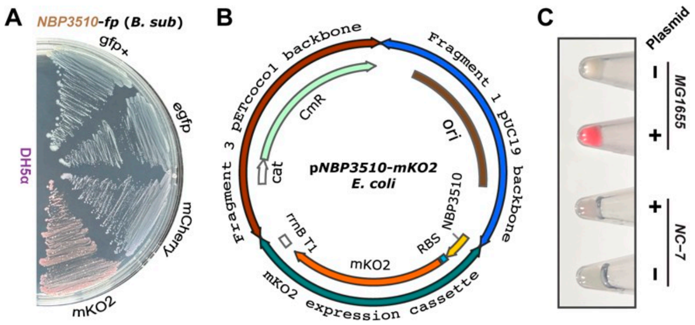

Figure S7. Construction and analysis of fluorescent expression strains of E. coli MG1655 and L-form NC-7. (A) E. coli DH5α strains expressing FPs from pNBP3510-gfp+/egfp/mCherry/mKO2 were grown on LB plate, where the bacteria colonies showed visible colors. (B) Construction of the plasmid pNBP3510-mKO2 for E. coli. (C) Cell pellets were collected from E. coli MG1655 and L-form NC-7 with/without pNBP3510-mKO2.

Table S1 List of primers used in this study

Name	Sequence (5’ to 3’)
Pspac F1	gtgatgataagtgggaaggac
Pspac R1	gctttccgtatgtagcatcac
Promoter F1	gactatattcaaaggtgaaagcactcg
Promoter R1	gtacctctagatgcttcaccacct
Promoter F2	gtttgtcggtgaacgctctctac
Promoter R2	cgcgatttccaatgaggttaagag
gfp+ R1	ccgtatgtagcatcaccttcacc
egfp R1	ccgtatgttgcatcaccttcac
staygold R1	ccatgacatcggtagttttccac
mCherry R1	cttggagccgtacatgaactgag
dsRed R1	ggaaggacagcttcatgtagtcg
tdTomato R1	catgttgttgtcctcggaggag
mKO2 R1	cgcgtagtgtcatctcttgatg
gfp+ F2	ccgttcaactagcagaccattatc
egfp F2	gcgttcaactagcagaccattatc
staygold F2	gatgtcccatatcactggattcg
mCherry F2	ctgaggtcaagaccacctacaagg
dsRed F2	caaccactacgtggactccaag
tdTomato F2	ctcctccgaggacaacaacatg
mKO2 F2	gattcttgaaatgccaggagacc
transform F3	cgcgaattatcaagctctgagc
transform R3	cgagggaagcgttcacagtttc
transform F4	catcattaatcatccttccaggg
transform R4	gctgaccgttagcgtttaagtac
HM-pUC19 F	gtgcccttaaacgccctgtcagaccaagtttactcat
HM-pUC19 R	gagaagtgcatgcttccagtcgggaaacctgtcgtg
HM-Ppro-mKO2-F	ccgactggaagcatgcacttctcaaagatccca
HM-Ppro-mKO2-R	gtgtgacgttctgcggccgctctagaaccggaac
HM-pETcoco-1-F	ctagagcggccgcagaacgtcacaccgtcagcag
HM-pETcoco-1-R	cttggtctgacagggcgtttaagggcaccaataactgcc
Fragment 3+1 F1	ctggcgattcaggttcatcatgc
Fragment 3+1 R1	gaagaacagtatttggtatctgcgctc
Fragment 1+2 F2	gcgttatcccctgattctgtgg
Fragment 1+2 R2	ctaagtcaaacgcgaaaggcattg
Fragment 2+3 F3	ggtcgacggtatcgataagcttg
Fragment 2+3 R3	cttacgtgccgatcaacgtctc
